# Association of Septal Myectomy With Quality of Life in Patients With Left Ventricular Outflow Tract Obstruction From Hypertrophic Cardiomyopathy

**DOI:** 10.1001/jamanetworkopen.2022.7293

**Published:** 2022-04-14

**Authors:** Milind Y. Desai, Albree Tower-Rader, Natalie Szpakowski, Amgad Mentias, Zoran B. Popovic, Nicholas G. Smedira

**Affiliations:** 1 The Hypertrophic Cardiomyopathy Center, Heart and Vascular Institute, Cleveland Clinic, Cleveland, Ohio; 2 Department of Cardiovascular Medicine, Massachusetts General Hospital, Boston

## Abstract

This cohort study assesses the association of septal myectomy with quality of life in patients with left ventricular outflow tract obstruction from hypertrophic cardiomyopathy.

## Introduction

In patients with obstructive hypertrophic cardiomyopathy (OHCM), surgical myectomy (SM) alleviates dynamic left ventricular outflow tract (LVOT) obstruction and relieves intractable symptoms.^1^ However, its association with patient-reported quality of life (QOL) and functional capacity has not been prospectively studied, to our knowledge. We describe the primary results of the Quality of Life and Functional Capacity Following Septal Myectomy in Obstructive Patients With Hypertrophic Cardiomyopathy (SPIRIT-HCM) study, a prospective investigator-initiated single-arm study (ClinicalTrials.gov identifier: NCT03092843).

## Methods

This cohort study was approved by the Cleveland Clinic, Cleveland, Ohio, institutional review board, and all patients provided written informed consent. This report follows the Strengthening the Reporting of Observational Studies in Epidemiology (STROBE) reporting guideline for cohort studies. The study protocol is provided in the Supplement.

Patients with symptomatic OHCM^2^ who were scheduled to undergo SM within 90 days of evaluation at Cleveland Clinic between March 2017 and June 2020 were prospectively enrolled. Clinical data were recorded at baseline and after a mean (SD) of 12 (6) months follow-up. All patients underwent comprehensive echocardiograms, including maximal LVOT obstruction (resting rate with or without provocation using Amyl nitrite, Valsalva, or exercise) and mitral regurgitation.^2^ Surgical procedures (SM with or without mitral valve or papillary muscle surgery) were recorded. Kansas City Cardiomyopathy Questionnaire (KCCQ) scores (ie, summary, physical limitation, symptoms, QOL, and social limitations) were recorded at baseline and follow-up, with scores ranging from 0 to 100, with higher score indicating better outcome.^3^ KCCQ summary scores were further stratified as 0 to 24, poor; 25 to 49, fair; 50 to 74, good; and 75 to 100, excellent.^3^ Overall increases in KCCQ scores were recorded. The primary end point was the proportion of patients with at least 5-point increases in KCCQ summary score. Results of a 6-minute walk test were also recorded. Perioperative stroke and mortality were recorded.

McNemar test was used to compare the change in categorical variables from baseline to follow up. To compare the change in continuous variables, paired *t* test (for normal distribution) or signed rank test (for on-normal distribution) were used. *P* values were 2-sided, and *P* < .001 was considered significant.

## Results

We enrolled 173 patients with OHCM (mean [SD] age, 52 (11) years; 107 [62%] men) who underwent SM, including 121 patients (70%) who underwent isolated SM and 52 patients (30%) who underwent SM with mitral valve or papillary muscle surgery. There was 1 death (3 weeks after procedure), no perioperative strokes, and 5 patients (3%) needed a pacemaker. At a median (IQR) of 14 (12-16) months after SM, 136 patients (79%) completed follow-up. Of these, 125 patients (92%) achieved the primary end point, while 109 patients (80%) experienced a large (>20 points) increase, 6 patients (4%) had a reduction, and 5 patients (4%) had no change in KCCQ summary score. An increase of at least 5 points in KCCQ summary score was observed in 54 of 62 patients (87%) in baseline New York Heart Association (NYHA) Class II and 71 of 74 patients (96%) in NYHA Class III or IV (*P* = .40). Also, 117 patients (86%) reported an excellent KCCQ summary score (≥75 points) at follow-up. Changes in several parameters from baseline to follow-up were significant (Table). Polar plots demonstrating significant increases in various KCCQ domain scores are shown in the Figure. There was also a significant increase in 6-minute walk test and a reduction in NT-pro BNP levels, along with LVOT gradients.

**Table.** Quality of Life, Functional Capacity, and Echocardiographic Data at Baseline and After Surgery

Variable	No. (%) (N = 136)		Difference (95% CI)	*P* value
	Baseline	Follow-up		
KCCQ Summary score, median (IQR), points	50 (36 to 66)	96 (86 to 100)	38 (35 to 42)	<.001
Category				
Very poor to poor (0-24 points)	14 (10)	2 (2)	NA	<.001
Poor to fair (25-49 points)	53 (39)	7 (5)	NA	
Fair to good (50-75 points)	47 (33)	10 (7)	NA	
Good to excellent (76-100 points)	23 (17)	117 (86)	NA	
KCCQ score, median (IQR), points				
Physical limitation	67 (53 to 83)	92 (90 to 100)	28 (24 to 32)	<.001
Symptoms	61 (40 to 79)	96 (80 to 100)	35 (19 to 31)	<.001
Quality of life	25 (13 to 38)	94 (75 to 100)	57 (52 to 61)	<.001
Social limitation	50 (25 to 75)	100 (92 to 100)	41 (36 to 46)	<.001
NYHA class				
I	0	91 (67)	NA	<.001
II	62 (46)	41 (30)	NA	
III/IV	74 (54)	4 (3)	NA	
6-min walk distance, median (IQR), m	370 (305 to 440)	436 (382 to 479)	67 (51 to 84)	<.001
Borg score, median (IQR), points				
Dyspnea	4 (2 to 6)	0 (0 to 3)	−2.9 (−3.4 to 2.4)	<.001
Fatigue	4 (1 to 7)	0 (0 to 3)	−2.4 (−3.1 to 1.8)	<.001
Peak LV outflow tract gradient provoked, median (IQR), mm Hg	100 (87 to 129)	13 (10 to 19)	−94 (−101 to −86)	<.001
Moderate or worse mitral regurgitation	61 (45)	9 (7)	NA	<.001
NT proBNP, median (IQR), pg/dL^a^	485 (191 to 1192)	267 (140 to 476)	−219 (−508 to −171)	<.001

Abbreviations: KCCQ, Kansas City Cardiomyopathy Questionnaire; LV, left ventricle; NA, not applicable; NT proBNP, N-terminal pro–brain natriuretic peptide; NYHA, New York Heart Association.

SI conversion factor: To convert NT proBNP to nanograms per liter, multiply by 1.

^a^ Measured in 87 patients.

**Figure.**
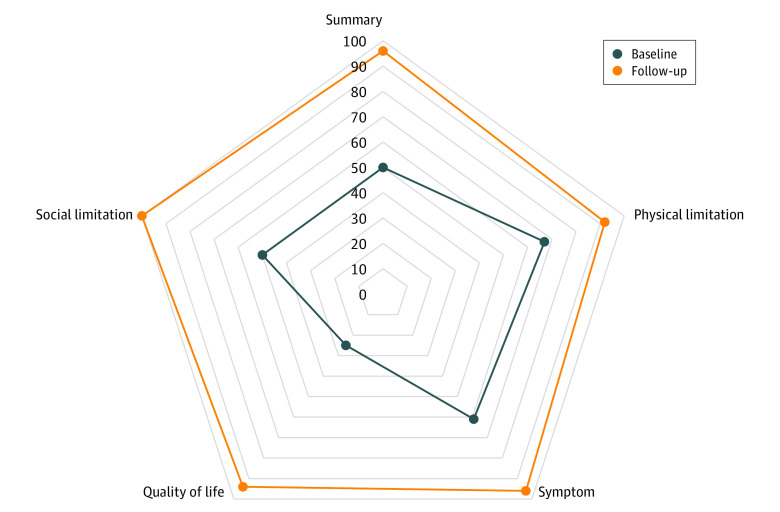
Polar Chart of Kansas City Cardiomyopathy Questionnaire Domain Scores From Baseline to Follow-up

## Discussion

In this cohort study, patients with symptomatic OHCM reported a significant increase in patient-reported QOL after SM, including 80% of patients demonstrating an increase of more than 20 points in KCCQ summary score. The increase in KCCQ summary score was significantly greater than what has been previously published for various diseases, including OHCM.^4,5,6^

This study was limited as a 1-group study conducted at a single tertiary care center with only 1 year of follow-up. Given the center’s expertise in SM, our results might not be generalizable across all centers.^4^ Furthermore, 8% of patients did not have improved KCCQ score, likely owing to an underrecognized phenotype with advanced disease (eg, diastolic dysfunction), obesity, and deconditioning associated with long-term restriction in physical activity.

## Conclusions

This prospective cohort study found that SM in patients with symptomatic OHCM significantly increased overall QOL and functional capacity. Whether this is maintained over the long-term remains to be determined.

## Notes

Supplement.
Study Protocol
