# Supplementary material for: Association of Septal Myectomy With Quality of Life in Patients With Left Ventricular Outflow Tract Obstruction From Hypertrophic Cardiomyopathy
Source: JAMA Netw Open. 2022 Apr 14;5(4):e227293. doi: 10.1001/jamanetworkopen.2022.7293 (PMC9011128; doi:10.1001/jamanetworkopen.2022.7293)
Supplement: Supplement. — Study Protocol [file jamanetwopen-e227293-s001.pdf]

Functional capacity and quality of life following septal myectomy in patients with obstructive hypertrophic cardiomyopathy

Principal Investigator:
Milind Desai, MD

Amendment 1 version: October 10, 2017
Original Version: January 25, 2017

SIGNATURE PAGE

The signature below constitutes the approval of this protocol and the attachments, and provides the necessary assurances that this trial will be conducted according to all stipulations of the protocol, including all statements regarding confidentiality, and according to local legal and regulatory requirements and applicable US federal regulations and ICH guidelines.

Principal Investigator

Signed: ____________________________________ Date: ________________

Name
Title

Table of Contents

page

Signature Page..i
List of Abbreviations..iii
Protocol Summary...iv

1 Key Roles..5
2 Background Information and Scientific Rationale....................6
  2.1 Background Information...6
  2.2 Rationale..7
  2.3 Potential Risks and Benefits...................................7
    2.3.1 Potential Risks..7
    2.3.2 Known Potential Benefits...................................7
3 Objectives...8
4 Study Design...9
  4.1 Selection of the Study Population..............................9
  4.2 Inclusion/Exclusion Criteria...................................9
5 Study Procedures/Evaluations.......................................10
  5.1 Study Procedures...10
  5.2 Subject Compensation...10
6 Study Schedule...11
  6.1 Screening..11
  6.2 Baseline visit...11
  6.3 Follow-up Visit..11
7 Assessment of Outcome Measures.....................................12
  7.1 Specification of the Appropriate Outcome Measures..............12
    7.1.1 Primary Outcome Measures...................................12
    7.1.2 Secondary Outcome Measures.................................12
8 Statistical Considerations...13
  8.1 Study Outcome Measures...13
  8.2 Sample Size Considerations.....................................13
  8.3 Participant Enrollment and Follow-Up...........................13
  8.4 Analysis Plan..13
9 Data Handling and Record Keeping...................................15
  9.1 Data Capture Methods...15
  9.2 Types of Data..15

Literature...17

List of Abbreviations

DASI	Duke Activity Status Index
ECG	Electrocardiogram
EF	Ejection Fraction
HCM	Hypertrophic Cardiomyopathy
HF	Heart Failure
ICD	Implantable Cardioverter-Defibrillator
ICF	Informed Consent Form
KCCQ	Kansas City Cardiomyopathy Questionnaire
LV	Left Ventricle
LVAD	Left Ventricular Assist Device
LVH	Left Ventricular Hypertrophy
LVOT	Left Ventricular Outflow Tract
METS	Metabolic Equivalents
NSVT	Nonsustained Ventricular Tachycardia
NYHA	New York Heart Association
PI	Principal Investigator
PROMIS	Patient-Reported Outcomes Measurement Information System
SAM	Systolic Anterior Motion
SCD	Sudden Cardiac Death
VO2	Oxygen Uptake
VT	Ventricular Tachycardia
VF	Ventricular Fibrillation

Protocol Summary

**Title**:	Functional capacity and quality of life following septal myectomy in patients with obstructive hypertrophic cardiomyopathy
**Population**:	175 patients, male and female, age 18-65, diagnosed with hypertrophic cardiomyopathy
**Number of Sites**:	1
**Study Duration**:	3 years
**Subject Duration**:	One visit prior to septal myectomy and one follow-up visit approximately 12±6 months following surgery per subject.
**Objectives**:	The primary aim of this study is to examine change in quality of life as measured by the KCCQ following septal myectomy in patients with obstructive hypertrophic cardiomyopathy as compared to prior to surgery. Secondary aims include the assessing change in functional capacity utilizing six-minute walk test distance, and quality of life as measured by the PROMIS and DASI following septal myectomy in patients with obstructive hypertrophic cardiomyopathy as compared to prior to surgery. The aim of the substudy is to examine change in exercise capacity by cardiometabolic exercise testing in a subset of patients who underwent cardiometabolic exercise testing prior to septal myectomy as part of their routine clinical care.

1 KEY ROLES

Individuals:

**Principal Investigator**	Milind Desai, MD
**Additional Investigators**	Albree Tower-Rader, MD
	Harry Lever, MD
	Nicholas Smedira, MD
	Maran Thamilarasan, MD
	Zoran Popovic, MD

Recruiting Site: Cleveland Clinic Main Campus

2 BACKGROUND INFORMATION AND SCIENTIFIC RATIONALE

2.1 Background Information

Hypertrophic cardiomyopathy is a common inherited cardiac condition with an incidence estimated to be approximately 1 in 500 and variable phenotypes (1). Symptoms of palpitations, chest pain, shortness of breath, heart failure and pre-syncope or syncope have been attributed to multiple aspects of the disease including left ventricular outflow tract (LVOT) obstruction, mitral regurgitation, diastolic dysfunction and atrial fibrillation. Approximately 70% of patients with HCM have either resting or provocable LVOT obstruction (2-4). Treatment for patients who are refractory to medical therapy has focused on the relief of LVOT obstruction either by septal myectomy or alcohol septal ablation (5-10).

Several reports of outcomes following septal myectomy have demonstrated improvements in symptoms. Additionally in one cohort, patients were shown to have similar mortality to the general population matched for age and gender (11). However, studies examining improvement following surgery have been limited to description of mortality rates and change in LVOT gradients and severity of mitral regurgitation. Symptoms have been qualitatively studied either as patients reporting yes/no to the presence of specific symptoms, or physician assessment of functional capacity as measured by New York Heart Association (NYHA) functional class (7,10,12-17). More recent studies evaluating the efficacy of alternative treatments like ventricular pacing have demonstrated qualitative improvements in symptoms (NYHA functional class, Minnesota Living with Heart Failure Questionnaire scores) as well as quantitatively with walk distances on six minute walk tests and peak oxygen uptake (VO2) by cardiometabolic exercise testing (18,19). One small study compared walk distance and peak oxygen uptake (VO2) in 20 patients with obstructive HCM who underwent septal myectomy and 19 patients who underwent pacemaker implantation and found improvement in the septal myectomy group, though this was a small study (20).

Quality of Life assessment tools have gained traction in their use more recently as an additional outcome metric. KCCQ is a disease-specific quality of life questionnaire which was designed and validated in the heart failure population (21-23), though it has been validated for use in patients with aortic stenosis (24). There is one study of a cohort of patients with HCM and been shown to correlate with peak oxygen uptake and inversely correlate with NYHA functional class at a cross-section in time (25). Though the Patient-Reported Outcomes Measurement Information System (PROMIS) has not been studied in a population with hypertrophic cardiomyopathy specifically, it was designed as a general health-related quality of life assessment tool and tested in a large patient cohort including patients with cardiac conditions, and shown to correlate well with other general health-related quality of life assessment tools like the EuroQol five dimensions (EQ-5D) questionnaire (26,27). Additionally the Duke Activity Status Index has been shown to correlate well with functional capacity and yield prognostic

value in patients with heart failure (28-30). Based on their prior use in patients with heart failure we chose to use these health-related quality of life metrics since patients who are symptomatic from LVOT obstruction in HCM often have symptoms found in heart failure such as functional limitation, dyspnea on exertion and heart failure.

2.2 Rationale

Currently septal myectomy is the preferred treatment for patients with LVOT obstruction and symptoms despite medical therapy though there have been few large studies evaluating quantitative improvements in functional capacity and quality of life following septal myectomy. This prospective cohort will address what quantitative improvements in functional capacity and quality of life may be seen following septal myectomy, and more importantly may serve to identify a cohort of patients whom do not benefit from surgery.

2.3 Potential Risks and Benefits

2.3.1 Potential Risks

Six-minute Walk Test

The risk of a six minute walk test are equivalent to those of normal walking.

Quality of Life Surveys

Questions composing these surveys may at times make patients feel embarrassed.

Cardiometabolic Stress Testing

During cardiometabolic stress testing patients breathe through a tube designed to quantify oxygen and carbon dioxide exchange. This portion of the test may make patients feel uncomfortable or claustrophobic. Additionally the process of exercising to peak exercise stress may make patients symptomatic or cause them distress.

2.3.2 Known Potential Benefits

There are no specific benefits to the patient.

3 OBJECTIVES

The primary aim of this study is to examine change in quality of life as measured by the KCCQ following septal myectomy in patients with obstructive hypertrophic cardiomyopathy as compared to prior to surgery. Secondary aims include the assessing change in functional capacity utilizing six-minute walk test distance, and quality of life as measured by the PROMIS and DASI following septal myectomy in patients with obstructive hypertrophic cardiomyopathy as compared to prior to surgery. The aim of the substudy is to examine change in exercise capacity by cardiometabolic exercise testing in a subset of patients who underwent cardiometabolic exercise testing prior to septal myectomy as part of their routine clinical care.

The study will be powered to detect a mild change in Kansas City Cardiomyopathy Questionnaire summary score since this disease-specific health-related quality of life assessment tool has been well studied and changes in clinical status have been validated within a heart failure population. A substudy will also be powered to detect an absolute increase in peak oxygen uptake on cardiometabolic exercise testing. Additional secondary end-points will include change in score on the Patient-Reported Outcomes Measurement Information System (PROMIS) global health questionnaire and Duke Activity Status Index (DASI), distance walked on six-minute walk test, and rate of atrial fibrillation, stroke, hospitalization for heart failure, repeat septal myectomy, left ventricular assist device or heart transplant, aborted sudden death including appropriate internal cardioverter defibrillator, or death by follow up visit.

4 STUDY DESIGN

4.1 Selection of the Study Population

Patient characteristics:

175 patients will be recruited

Recruiting locations:

Outpatient setting from Cleveland Clinic Main Campus

Eligibility criteria:

Patients aged 18-65 with an established diagnosis of obstructive HCM defined as LVH defined as any segment ≥15mm thick, without a predisposing cause, and left ventricular outflow tract gradient ≥50mmHg at rest or with provocation scheduled to undergo septal myectomy within 90 days following the baseline encounter.

Patients are eligibile to take part in the substudy if they have a **Cardiometabolic Stress Testing** done within 6 months of their scheduled surgery.

4.2 Inclusion/Exclusion Criteria

Inclusion criteria:

- Patients aged 18-65 with an established diagnosis of obstructive HCM defined as LVH defined as any segment ≥15mm thick, without a predisposing cause, and left ventricular outflow tract gradient ≥50mmHg at rest or with provocation scheduled to undergo septal myectomy within 90 days following the baseline encounter.
- A subset of patients who underwent cardiometabolic exercise testing as part of their testing within 6 months prior to their scheduled septal myectomy, will be eligible for a substudy examining improvements in performance on cardiometabolic exercise testing following septal myectomy

Exclusion criteria:

1) Prior septal myectomy or alcohol septal ablation
2) Prior myocardial infarction
3) Uncontrolled arrhythmias including ventricular tachycardia and atrial fibrillation
4) Uncontrolled hypertension
5) Angiographically documented coronary stenosis >50%, if available at time of screening
6) Inability to provide informed consent
7) Inability to walk without assistive devices
8) Peripheral artery disease with claudication

5 STUDY PROCEDURES/EVALUATIONS

5.1 Study Procedures

- **Intake history, physical, and recording of prior test results**

Per routine clinical care at baseline and follow up visits:

- A medical history will be obtained including a complete family history
- A limited physical examination will be performed including heart rate and blood pressure
- Results of testing prior to septal myectomy available at the baseline visit will be recorded including ECG, exercise testing, Holter monitoring, and echocardiography.
- ECG and echocardiography per standard of care at follow up visits

- **Study-specific Testing**

Six-minute walk test at baseline and follow-up visit

Quality of Life Assessments (KCCQ, PROMIS, and DASI) at baseline and follow-up visit

Cardiometabolic exercise testing at follow up in the substudy patients who had undergone cardiometabolic exercise testing prior to septal myectomy as part of their routine care

5.2 Subject Compensation

Compensation for parking for required visits for the study protocol will be compensated. Patients enrolled in the substudy may also receive compensation for a meal on the day of the cardiometabolic stress test. Testing performed outside of the scope of normal clinical practice including 6-minute walk test and quality of life assessments (KCCQ, PROMIS and DASI) at baseline and follow up visit, and cardiometabolic exercise testing at follow up visit in the substudy will be covered in full by the study.

6 STUDY SCHEDULE

6.1 Screening

Informed consent must be obtained by a member of the study team prior to any study-related procedures. Patient's medical records will be reviewed to be certain of diagnosis and eligibility. Patients will be identified for eligibility for the substudy evaluating change in performance on cardiometabolic exercise testing simultaneously.

6.2 Baseline visit

Initial encounter for the research protocol will involve a six-minute walk test and quality of life assessments prior to septal myectomy. Clinical data regarding the baseline visit (history, physical exam, echocardiogram, cardiometabolic stress test, etc) will be obtained by the individual physician per standard of care.

6.3 Follow-up Visit

Follow up visit will be scheduled at a mean of 12±6 months post-septal myectomy for a six-minute walk test and quality of life assessments will be administered for a second time. For the subset of patients who underwent cardiometabolic exercise testing prior to septal myectomy who are enrolled in the substudy, they will undergo repeat cardiometabolic exercise testing again at this point. Post-operative clinical follow up data will be obtained from the outpatient visit per standard of care, and data collection regarding outcomes (atrial fibrillation, stroke, ICD discharge etc.) will terminate at the time of the follow up visit.

7 ASSESSMENT OF OUTCOME MEASURES

7.1 Specification of the Appropriate Outcome Measures

7.1.1 Primary Outcome Measures

Primary outcome measure is improvement in:

- Kansas City Cardiomyopathy Questionnaire (KCCQ) summary score

7.1.2 Secondary Outcome Measures

Secondary outcome measures will include:

- Kansas City Cardiomyopathy Questionnaire (KCCQ) summary score
- Patient-Reported Outcomes Measurement Information System (PROMIS) global health score
- Duke Activity Status Index (DASI) score
- Distance walked with 6-minute walk test
- Peak oxygen uptake on cardiometabolic exercise testing
- Death
- Aborted sudden cardiac death including appropriate internal cardioverter defibrillator discharge
- Need for left ventricular assist device or heart transplantation
- Need for repeat septal myectomy
- Atrial fibrillation
- Stroke
- Hospitalization for heart failure
- Cardiac Rehabilitation post-surgery

8 STATISTICAL CONSIDERATIONS

8.1 Study Outcome Measures

The primary endpoint of this prospective study is a mild improvement in Kansas City Cardiomyopathy summary score (increase in 5).

Secondary endpoints include improvement in Kansas City Cardiomyopathy Questionnaire (KCCQ) summary score, Patient-Reported Outcomes Measurement Information System (PROMIS) global health score, Duke Activity Status Index (DASI) score, Distance walked with 6-minute walk test, all-cause mortality, ventricular tachyarrhythmias, left ventricular assist device placement, heart transplantation, repeat septal myectomy, hospitalization for heart failure, atrial fibrillation, and stroke.

These will be assessed at patient follow up visit.

8.2 Sample Size Considerations

Assuming an average KCCQ summary score of 76±20 in patients with hypertrophic cardiomyopathy based on previously published literature (25) and an alpha error rate of 5%, 133 patients provides over 90% power to detect a mild change in KCCQ summary score defined as an absolute change of 5 points. An additional 42 patients (175 total) will be enrolled to compensate for a projected dropout rate of 10% per year over three years.

For the substudy, assuming an average maximal oxygen uptake (peak VO2) of 19.4 ± 6.4 ml/kg/min based on prior studies in patients with obstructive hypertrophic cardiomyopathy prior to septal myectomy (20) and an alpha error rate of 5%, 32 patients provides over 90% power to detect an absolute change of 2 ml/kg/min. An additional 3 patients (35 total) will be enrolled in the substudy to compensate for an absolute dropout rate of 10%.

8.3 Participant Enrollment and Follow-Up

175 patients will be followed through follow up visit at 12±6 months post-septal myectomy.

8.4 Analysis Plan

Overall statistical analysis should be straightforward and involve paired t-tests comparing the change in scores on quality of life assessments, and distance walked on six-minute walk tests. Additionally the incidence of other outcomes including atrial fibrillation, hospitalization for heart failure, repeat septal myectomy, sudden cardiac death or appropriate ICD discharge will be noted. For patients in the substudy, paired t-tests will also be used to compare peak oxygen

uptake prior to and following septal myectomy. Additional analyses examining the correlation of scores on various quality of life assessments to performance on exercise testing as measured by distance walked on six-minute walk test and peak oxygen uptake in the substudy patients may also yield valuable information regarding the performance of these assessments in the HCM population.

9 DATA HANDLING AND RECORD KEEPING

9.1 Data Capture Methods

All clinical and intake data will be captured on electronic data collection forms (RedCap).

9.2 Types of Data

4 types of data will be collected:

- Clinical intake and follow-up data
- Six-minute walk test data
- Quality of Life Assessments (KCCQ, PROMIS and DASI) data
- Cardiometabolic exercise testing data (for substudy)

Literature

1. Maron BJ, Gardin JM, Flack JM, Gidding SS, Kurosaki TT, Bild DE. Prevalence of hypertrophic cardiomyopathy in a general population of young adults. Echocardiographic analysis of 4111 subjects in the CARDIA Study. Coronary Artery Risk Development in (Young) Adults. Circulation 1995;92:785-9.
2. Maron MS, Olivotto I, Betocchi S et al. Effect of left ventricular outflow tract obstruction on clinical outcome in hypertrophic cardiomyopathy. N Engl J Med 2003;348:295-303.
3. Maron MS, Olivotto I, Zenovich AG et al. Hypertrophic cardiomyopathy is predominantly a disease of left ventricular outflow tract obstruction. Circulation 2006;114:2232-9.
4. Elliott PM, Gimeno JR, Tome MT et al. Left ventricular outflow tract obstruction and sudden death risk in patients with hypertrophic cardiomyopathy. Eur Heart J 2006;27:1933-41.
5. Maron BJ. Hypertrophic cardiomyopathy: a systematic review. Jama 2002;287:1308-20.
6. Spirito P, Seidman CE, McKenna WJ, Maron BJ. The management of hypertrophic cardiomyopathy. N Engl J Med 1997;336:775-85.
7. McCully RB, Nishimura RA, Tajik AJ, Schaff HV, Danielson GK. Extent of clinical improvement after surgical treatment of hypertrophic obstructive cardiomyopathy. Circulation 1996;94:467-71.
8. Yu EH, Omran AS, Wigle ED, Williams WG, Siu SC, Rakowski H. Mitral regurgitation in hypertrophic obstructive cardiomyopathy: relationship to obstruction and relief with myectomy. J Am Coll Cardiol 2000;36:2219-25.
9. Sigwart U. Non-surgical myocardial reduction for hypertrophic obstructive cardiomyopathy. Lancet 1995;346:211-4.
10. Qin JX, Shiota T, Lever HM et al. Outcome of patients with hypertrophic obstructive cardiomyopathy after percutaneous transluminal septal myocardial ablation and septal myectomy surgery. J Am Coll Cardiol 2001;38:1994-2000.
11. Ommen SR, Maron BJ, Olivotto I et al. Long-term effects of surgical septal myectomy on survival in patients with obstructive hypertrophic cardiomyopathy. J Am Coll Cardiol 2005;46:470-6.
12. ten Berg JM, Suttorp MJ, Knaepen PJ, Ernst SM, Vermeulen FE, Jaarsma W. Hypertrophic obstructive cardiomyopathy. Initial results and long-term follow-up after Morrow septal myectomy. Circulation 1994;90:1781-5.
13. Cohn LH, Trehan H, Collins JJ, Jr. Long-term follow-up of patients undergoing myotomy/myectomy for obstructive hypertrophic cardiomyopathy. Am J Cardiol 1992;70:657-60.
14. Robbins RC, Stinson EB. Long-term results of left ventricular myotomy and myectomy for obstructive hypertrophic cardiomyopathy. J Thorac Cardiovasc Surg 1996;111:586-94.
15. Heric B, Lytle BW, Miller DP, Rosenkranz ER, Lever HM, Cosgrove DM. Surgical management of hypertrophic obstructive cardiomyopathy. Early and late results. J Thorac Cardiovasc Surg 1995;110:195-206; discussion 206-8.
16. Williams WG, Wigle ED, Rakowski H, Smallhorn J, LeBlanc J, Trusler GA. Results of surgery for hypertrophic obstructive cardiomyopathy. Circulation 1987;76:V104-8.
17. Maron BJ, Merrill WH, Freier PA, Kent KM, Epstein SE, Morrow AG. Long-term clinical course and symptomatic status of patients after operation for hypertrophic subaortic stenosis. Circulation 1978;57:1205-13.

18. Lenarczyk R, Wozniak A, Kowalski O et al. Effect of cardiac resynchronization on gradient reduction in patients with obstructive hypertrophic cardiomyopathy: preliminary study. Pacing Clin Electrophysiol 2011;34:1544-52.
19. Berruezo A, Vatasescu R, Mont L et al. Biventricular pacing in hypertrophic obstructive cardiomyopathy: a pilot study. Heart Rhythm 2011;8:221-7.
20. Ommen SR, Nishimura RA, Squires RW, Schaff HV, Danielson GK, Tajik AJ. Comparison of dual-chamber pacing versus septal myectomy for the treatment of patients with hypertrophic obstructive cardiomyopathy: a comparison of objective hemodynamic and exercise end points. J Am Coll Cardiol 1999;34:191-6.
21. Green CP, Porter CB, Bresnahan DR, Spertus JA. Development and evaluation of the Kansas City Cardiomyopathy Questionnaire: a new health status measure for heart failure. J Am Coll Cardiol 2000;35:1245-55.
22. Spertus J, Peterson E, Conard MW et al. Monitoring clinical changes in patients with heart failure: a comparison of methods. Am Heart J 2005;150:707-15.
23. Heidenreich PA, Spertus JA, Jones PG et al. Health status identifies heart failure outpatients at risk for hospitalization or death. J Am Coll Cardiol 2006;47:752-6.
24. Arnold SV, Spertus JA, Lei Y et al. Use of the Kansas City Cardiomyopathy Questionnaire for monitoring health status in patients with aortic stenosis. Circ Heart Fail 2013;6:61-7.
25. Huff CM, Turer AT, Wang A. Correlations between physician-perceived functional status, patient-perceived health status, and cardiopulmonary exercise results in hypertrophic cardiomyopathy. Qual Life Res 2013;22:647-52.
26. Cella D, Riley W, Stone A et al. The Patient-Reported Outcomes Measurement Information System (PROMIS) developed and tested its first wave of adult self-reported health outcome item banks: 2005-2008. J Clin Epidemiol 2010;63:1179-94.
27. Revicki DA, Kawata AK, Harnam N, Chen WH, Hays RD, Cella D. Predicting EuroQol (EQ-5D) scores from the patient-reported outcomes measurement information system (PROMIS) global items and domain item banks in a United States sample. Qual Life Res 2009;18:783-91.
28. Grodin JL, Hammadah M, Fan Y, Hazen SL, Tang WH. Prognostic value of estimating functional capacity with the use of the duke activity status index in stable patients with chronic heart failure. J Card Fail 2015;21:44-50.
29. Parissis JT, Nikolaou M, Birmpa D et al. Clinical and prognostic value of Duke's Activity Status Index along with plasma B-type natriuretic peptide levels in chronic heart failure secondary to ischemic or idiopathic dilated cardiomyopathy. Am J Cardiol 2009;103:73-5.
30. Tang WH, Topol EJ, Fan Y et al. Prognostic value of estimated functional capacity incremental to cardiac biomarkers in stable cardiac patients. J Am Heart Assoc 2014;3:e000960.
